# Lateral vibration data of an 18-story timber-concrete hybrid building obtained by on-site vibration tests

**DOI:** 10.1016/j.dib.2023.109501

**Published:** 2023-08-18

**Authors:** Yuji Miyazu, Cristiano Loss

**Affiliations:** a Department of Architecture, Tokyo University of Science, 2641 Yamazaki, Noda, Chiba 278-8510, Japan; b Sustainable Engineered Structural Solutions Laboratory, Department of Wood Science, University of British Columbia, 2424 Main Mall, Vancouver, BC V6T 1Z4, Canada

**Keywords:** Microtremor, Human-powered excitation, Velocity, Hybrid timber structures, Tall mass timber

## Abstract

The dataset provided in this paper includes the lateral vibration data obtained by on-site vibration tests on an 18-story timber-concrete hybrid building, which was completed in 2017 and is located within the University of British Columbia campus in Vancouver, Canada. Aiming at evaluating vibration properties such as natural periods, damping ratios, and mode shapes, microtremor measurement (i.e., ambient vibration test) and human-powered excitation tests were conducted from March 27^th^ to 29^th^, 2023. Lateral velocity data of the building were collected using eight servo velocity sensors, type VSE-15D, and a data acquisition system, type SPC-52, composed of an A/D converter and a DC amplifier. The voltage data picked up by the sensors was first converted to digital data and then transferred to the hard disk drive in a laptop through Ethernet. In the microtremor measurements, the lateral vibration in the longitudinal and the transverse directions of the test building were measured with three types of locations of the sensors in order to evaluate the vibration behavior along the height of the building and its torsional behavior. The human-powered excitation tests were carried out by pushing walls by eight persons in accordance with the fundamental frequency in the transverse direction, aiming at obtaining larger amplitude than with microtremor. The dataset is useful to deepen the understanding of the dynamic properties of tall timber-concrete hybrid buildings and encourage reasonable structural design and health monitoring of tall mass timber buildings.

Specifications Table

**Table:** 

Subject	Civil and Structural Engineering
Specific subject area	This dataset suits structural dynamics, system identification, and structural health monitoring, with specific reference to tall timber-concrete hybrid buildings.
Type of data	Table Image Chart Graph Figure
How the data were acquired	Vibration tests were conducted by using velocity sensors, type VSE-15D [1], and a data acquisition system, type SPC-52 [2], which were produced by Tokyo Sokushin. The VSE-15D is the uniaxial servo velocity sensor which has a differentiator in a feedback circuit. The voltage data measured by the VSE-15D were converted to digital data through SPC-52, which consisted of a DC amplifier and a 24-bit A/D converter. The digital data were transferred from SPC-52 to a laptop PC with a 64-bit Microsoft Windows operating system through Ethernet. Eight velocity sensors were located on the floors of the building, and two-directional vibrations were measured by changing the direction of the sensors.
Data format	Raw Analyzed
Description of data collection	Microtremor measurement and human-powered excitation tests were conducted on an 18-story timber-concrete hybrid building located within the University of British Columbia Vancouver campus, Canada. The sampling rate was 200 Hz. The velocity data were collected in a hard disk drive through an SPC-52 Data Acquisition Software as Pwave files (.dbl), which were Tokyo Sokushin's original binary files. The Pwave files were converted to text files by Pwave32 software.
Data source location	• Institution: Sustainable Engineered Structural Solutions Laboratory, Department of Wood Science, University of British Columbia • City/Town/Region: Vancouver, British Columbia • Country: Canada • Latitude and longitude (and GPS coordinates, if possible) for collected samples/data: 49°16’10” N, 123°15’4” W
Data accessibility	Repository name: Mendeley Data Data identification number: DOI: 10.17632/rt7w3txv2z.1 Direct URL to data: https://data.mendeley.com/datasets/rt7w3txv2z/1

## 1 Value of the Data

• The prediction formulas of natural periods and damping ratios for hybrid timber-based tall buildings are absent in current building codes and literature, leading the structural design of such buildings to conservative and uneconomical patterns compared to their concrete and steel counterparts.
• The system's vibration properties, such as natural periods, damping ratios, and mode shapes, are essential information in the structural design and structural health monitoring, as well as for applying a performance-based design framework. The vibration data of buildings can be used to estimate the vibration properties; therefore, the data provided here are essential for encouraging reasonable and economical design of tall timber-based hybrid buildings.
• Authorities in charge of reviewing the building codes and design standards (e.g., Canadian Standards Association (CSA), American Society of Civil Engineers (ASCE), European Committee for Standardization (CEN), International Code Council (ICC) and more), civil engineering firms (e.g., Arup, SOM, Ramboll and more) and not-for-profit R&D organizations, such as FPInnovations, RISE and EMPA will benefit from this dataset. In addition, researchers, civil engineers and students involved in the research related to tall mass timber buildings, structural health monitoring and structural dynamics can find interest in using this dataset.
• This data can be compared with those obtained from other buildings to investigate the feature of this building and to deepen the understanding of vibration property of tall mass timber buildings.
• This dataset is set to be reused in future studies, whether when looking at modelling the lateral dynamic properties of tall buildings or analyzing the long-term dynamic variation of the case study building.
• This data can also be reused when researchers, who specialize in structural engineering, data science or computer science, validate their computing strategies or other post-processing numerical methods.

## 2 Objective

In recent years, tall timber-concrete hybrid buildings, which consist of structural systems made of mass timber products and concrete materials, are becoming alternatives to conventional steel or reinforced concrete tall buildings. According to a survey undertaken by the Council on Tall Buildings and Urban Habitat in 2022 [3], timber-concrete hybrid buildings account for about 60% of tall mass timber buildings which are over 50 m in height.

As emerging technologies, approaches used for their design may result in over-conservative solutions and produce acceptable structures only at the price of using advanced analysis methods, which require access to an actual dataset for their validation. Particularly, information on the lateral vibration properties of a building plays an important role in estimating its dynamic response accurately, whether considering low-magnitude loading scenarios due to wind or extreme loading scenarios, such as in areas prone to seismic hazards.

The lateral vibration properties can be identified through operational modal analysis, which is quite practical and sometimes the only way forward, especially when the building is in service. Therefore, this dataset was generated through on-site vibration tests on an actual tall timber-concrete hybrid building.

## 3 Data Description

**Fig. 1:**
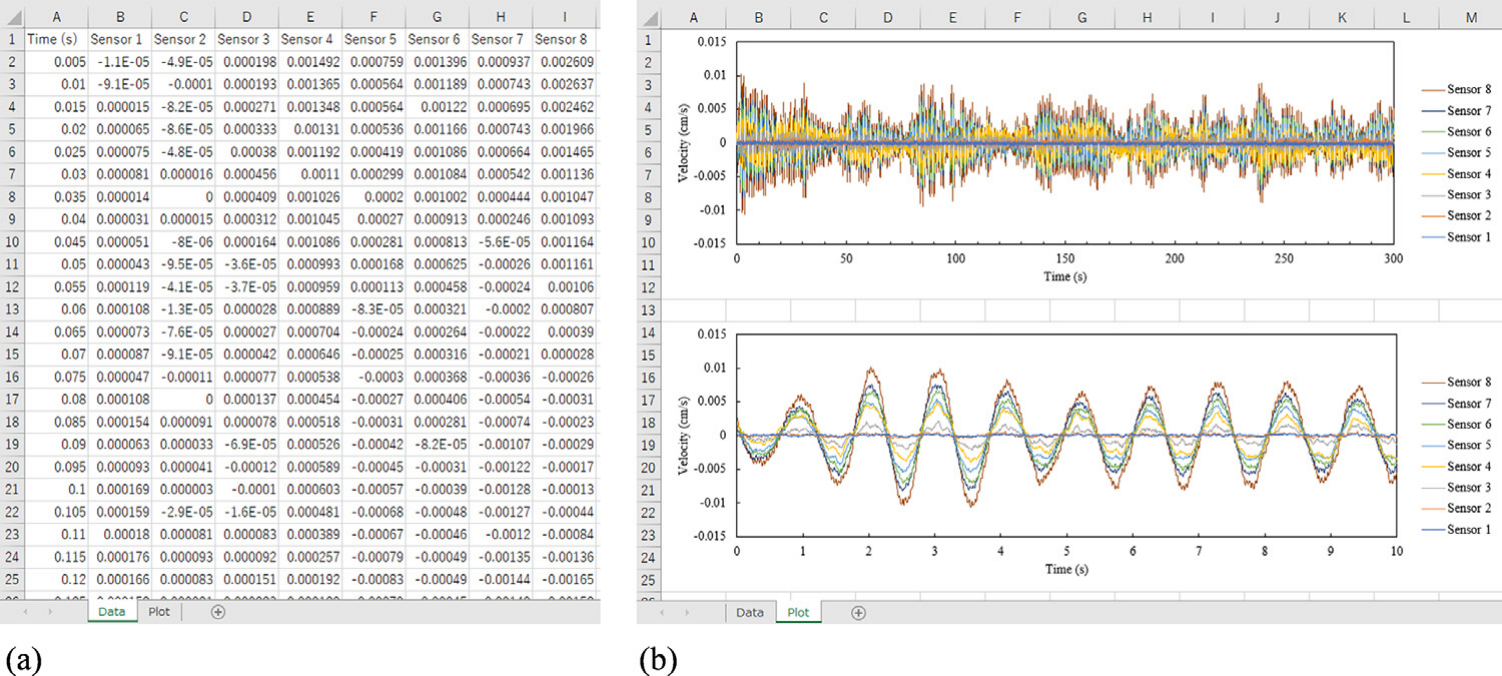
Composition of a spreadsheet file: (a) Data-sheet, (b) Plot-sheet.

The dataset provided in this article comprises six Microsoft Excel Spreadsheet (.xlsx) files. All the files have been uploaded to Mendeley Data [4]. Such files have collected the time-series vibration data acquired by servo velocity sensors and their velocity-time curves. Table 1 provides specifications of the data files provided in the repository. The data file named ‘Case 3_Y_free.xlsx’ contains the data obtained by human-powered excitation tests, while the other data files include the data acquired by microtremor measurements. Specifically, the location of sensors, definition of measurement direction, and sensor number and measured time are discussed in Sections 4.3 and 4.5. Each spreadsheet file contains two sheets named ‘Data’ and ‘Plot’ as shown in Fig. 1. The time-series velocity data are listed in the Data-sheet as presented in Fig. 1a, while the velocity-time curves are shown in the Plot-sheet as presented in Fig. 1b. Note that the velocity data picked up by Sensor No. 3 is not contained in Case 3_Y_free.xlsx because the data exceeded the assigned measurement range, as explained in Section 4.5.

**Table 1:** Specifications of data files in the repository.

File name	Location of sensors	Measurement direction	Number of sensors	Measured time
Case 1_X.xlsx	Case 1	X	8 (Sensor No. 1 - 8)	300 s
Case 1_Y.xlsx		Y	8 (Sensor No. 1 - 8)	300 s
Case 2_X.xlsx	Case 2	X	8 (Sensor No. 1 - 8)	300 s
Case 2_Y.xlsx		Y	8 (Sensor No. 1 - 8)	300 s
Case 3_Y.xlsx	Case 3	Y	8 (Sensor No. 1 - 8)	300 s
Case 3_Y_free.xlsx		Y	7 (Sensor No. 1, 2, 4 - 8)	60 s

**Fig. 2:**
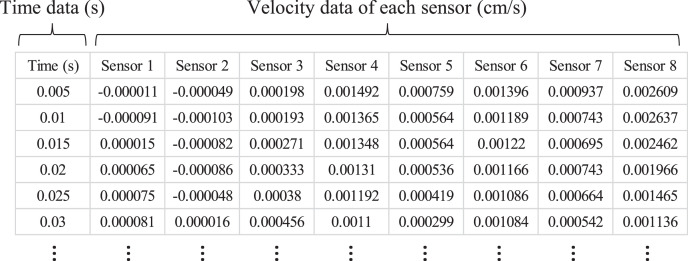
An example of time-series data excerpted from the Data-sheet of Case 1_X.xlsx.

**Fig. 3:**
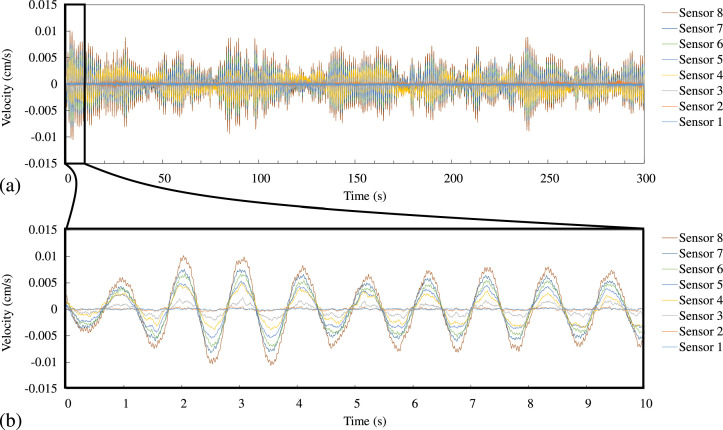
Velocity-time curves contained in the Plot-sheet of Case 1_X.xlsx: (a) Whole time, (b) During the first 10 s.

**Fig. 4:**
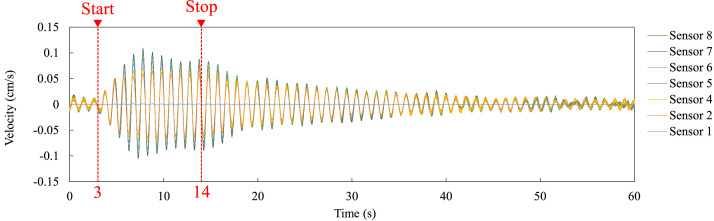
Velocity-time curve contained in the Plot-sheet of Case 3_Y_free.xlsx.

With specific reference to the content of each file, Fig. 2 shows an excerpt from the Data-sheet of Case 1_X.xlsx within the 0.005 to 0.03 s timeframe. In the first column, the time data in s, while the following columns contain velocity data recorded from sensors No. 1 to No. 8, and with the unit in cm/s. Fig. 3 reports the velocity-time curve obtained by one of the microtremor measurements, which is included in the Plot-sheet of Case 1_X.xlsx. Fig. 3b describes the enlarged plot during the first 10 s among the whole 300 s data shown in Fig. 3a. Fig. 4 illustrates the velocity-time curve in the Plot-sheet of Case 3_Y_free.xlsx, which was acquired by a free vibration test explained in the next section.

## 4 Experimental Design, Materials and Methods

### 4.1 Building Overview

**Fig. 5:**
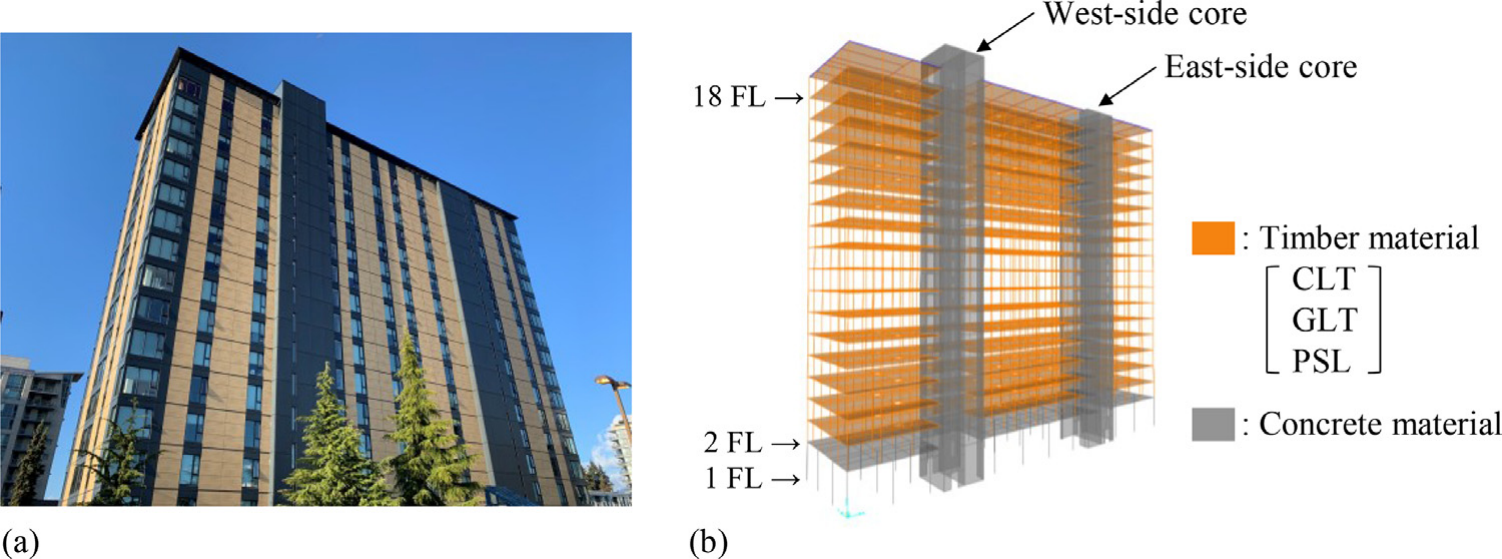
Tested building: (a) Exterior view, (b) 3D drawing of structural members.

The tested building is an 18-story timber-concrete hybrid residential building located within the University of British Columbia Vancouver campus, Canada [5]. Upon opening in 2017, it was immediately listed as the tallest timber hybrid building in the world by the Council on Tall Buildings and Urban Habitat. Fig. 5a shows the exterior view of the building from the south-west side. The footprint and the height of the building are 840 m^2^ (15 m × 56 m) and 53 m, respectively. This building is mainly composed of concrete materials and mass timber materials such as cross-laminated timber (CLT), glued laminated timber (GLT), and parallel strand lumber (PSL), as illustrated in Fig. 5b. The GLT and PSL elements are used for columns, whereas the CLT panels are used as floor slabs. Two reinforced concrete (RC) cores, which are used as staircases and elevator shafts, withstand the lateral loads caused by earthquakes and wind, while the timber columns transfer gravity loads to the 2^nd^ concrete podium. The CLT floor panels and their metal connections act as diaphragms, transferring lateral loads to the RC cores. Regarding to the timber floors, i.e., 2^nd^ to 18^th^ floors, the typical structural bay is 4 m × 2.85 m, and the floor-to-floor height is 2.8 m.

The concrete was designed and mixed in accordance with CSA A23.1 to develop a compressive strength of at least 35 MPa after 56 days. The CLT panel is 169 mm thick and has five layers consisting of two outer layers with E2 stress grade and three inner layers with V2 stress grade. The stress grades of the PSL and GLT columns are 2.2E and 16c-E, respectively. The cross-section of the columns is 265 mm × 265 mm in the lower levels and 265 mm × 215 mm in the upper levels.

### 4.2 Testing Equipment

Eight servo velocity sensors, type VSE-15D [1], were used to measure velocity in the lateral direction. The measurable frequency range of the sensor was from 0.2 Hz to 70 Hz, and the resolution of velocity was 1 × 10^−5^ cm/s. The sensitivity and the resistance of the calibration coil for the sensor were 6 × 10^−4^ A/m/s^2^ and 550 Ω ( ± 20%), respectively. The external dimensions of the sensor were 55 mm × 69.5 mm × 72 mm. The velocity sensors were connected by shielded cables, type NWS-1256 with EPRC05-P8F and EPRC05-P8M connectors, to the data acquisition systems, type SPC-52 [2], which consisted of a DC amplifier and a 24-bit A/D converter. The voltage data output from the sensor was converted to digital data by the data acquisition system; then the digital data was transferred to a laptop with a 64-bit Microsoft Windows operating system by Ethernet. The digital data were collected in a hard disk drive through a Data Acquisition Software (version 1.6.7235.32035) as Pwave files (.dbl), which were Tokyo Sokushin's original binary files; then the Pwave files were converted to text files by Pwave32 software (version 8.9.8). The sampling rate was 200 Hz in all the measurements. Both VSE-15D and SPC-52 were produced by Tokyo Sokushin Co., Ltd, and all the sensors were checked their accuracy and synchronization through preliminary tests.

### 4.3 Location of Sensors

**Fig. 6:**
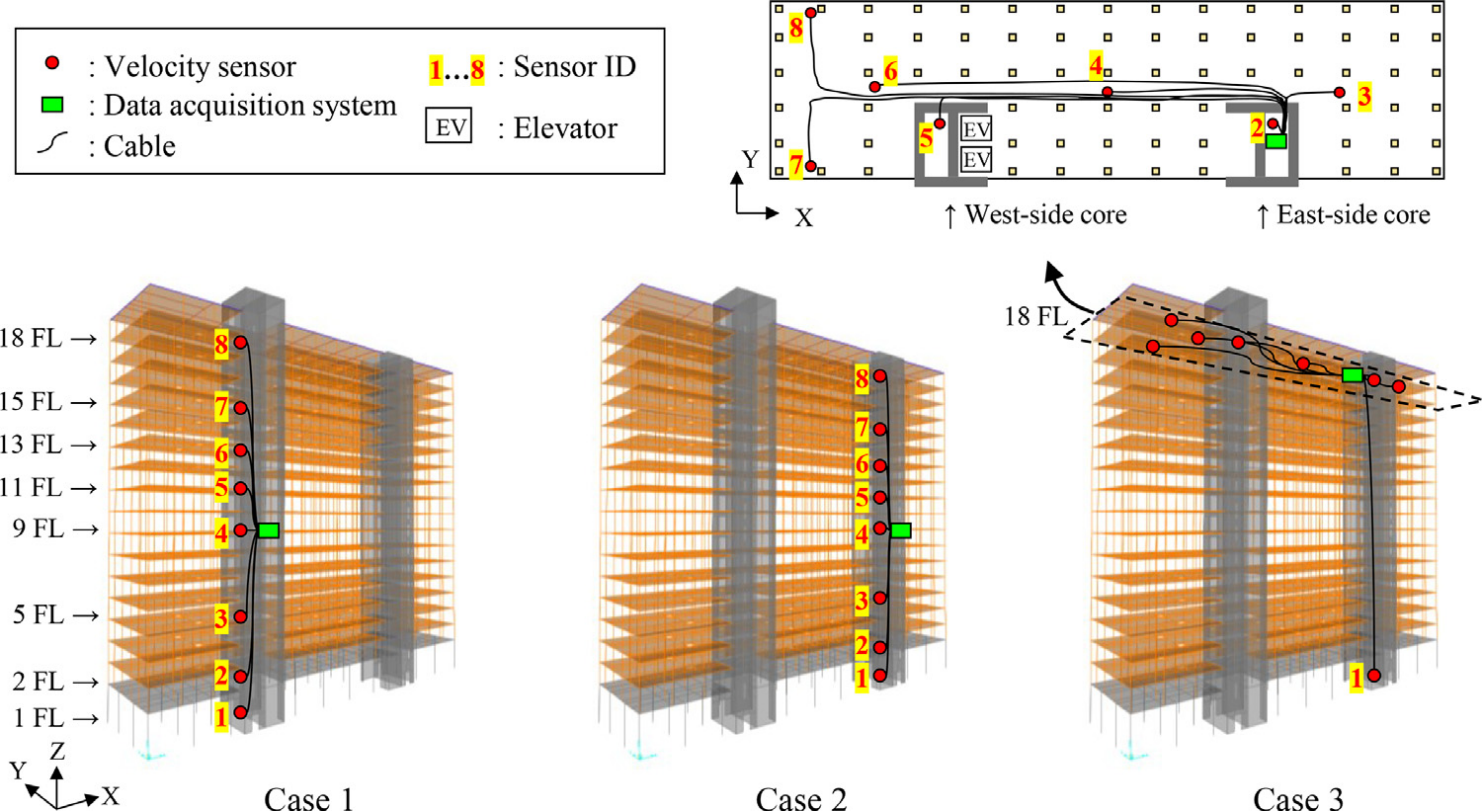
Location of velocity sensors in each testing case.

**Fig. 7:**
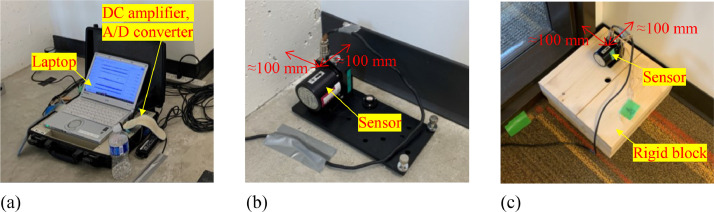
Setting of velocity sensors and data acquisition systems: (a) The data acquisition system, (b) The sensor in the RC core, (c) The sensor on the 18^th^ floor.

Fig. 6 shows the locations of the velocity sensors and the data acquisition system setup for testing Cases 1 to 3. In Cases 1 and 2, the sensors were located on the 1^st^, 2^nd^, 5^th^, 9^th^, 11^th^, 13^th^, 15^th^, and 18^th^ floors in the west-side core and the east-side core, respectively, to capture the vibration of the RC cores. In Case 3, one sensor was put on the 1^st^ floor and the others were placed on the 18^th^ floor aiming to catch the torsional vibration of the building and the in-plane deformation of the CLT floor diaphragm. The sensors and the data acquisition system are displayed in Fig. 7. The sensors in the RC cores were directly placed on the RC floor, while the sensors on the 18^th^ floor were mounted on rigid blocks to avoid an unstable setting caused by the flexibility of the carpet on the floor. The distance from the sensor to the walls or columns was set at 100 mm, as illustrated in Figs. 7b and c; however, it was moved within 300 mm from the walls or columns when the floor under the sensor was not flat.

### 4.4 Tuning of Sensors

The gain of the DC amplifier was set to 32 in order to ensure enough precision based on the on-site measurement conducted before the main test. After mounting all the sensors on the designated locations, velocity data were monitored for five to ten minutes to confirm that all the sensors were stable and ready to start measurement.

### 4.5 Testing Program

**Fig. 8:**
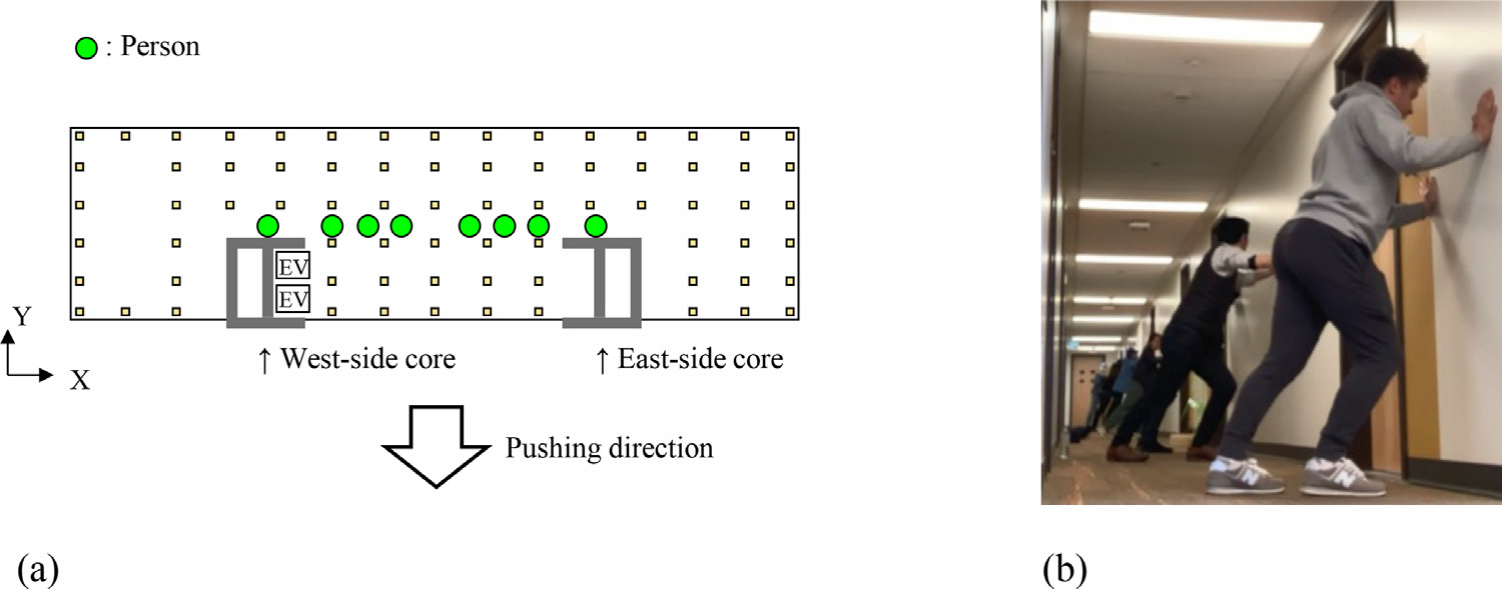
Human-powered excitation test: (a) The location of the persons, (b) An instant taken during testing.

The on-site test series were conducted sequentially on March 27^th^, 28^th^ and 29^th^, 2023. Table 2 summarizes the measurement method for each day. Two types of tests, microtremor measurements and human-powered excitation tests were conducted in this program. The microtremor measurements were carried out to acquire low-amplitude vibration (i.e., ambient vibration) caused by ambient excitations such as wind and traffic, while the human-powered excitation tests were performed to obtain larger amplitude than with microtremor. Measurement directions X and Y are defined in Fig. 6. Specifically, the X- and Y-directions correspond to the longitudinal and transversal directions of the building, respectively. In all the measurements, the elevator in the west-side RC core was turned off for 10 min to avoid generating vibration which would disturb the velocity data. The human-powered excitation test of Case 3 was carried out by pushing walls in the Y-direction as shown in Fig. 8. Eight persons were engaged in the test and arranged symmetrically to the center of the 18^th^ floor as illustrated in Fig. 8a, and they pushed walls by moving their body in accordance with the fundamental frequency of 1 Hz which was evaluated via post-processing data analysis of the microtremor measurements. The human-powered excitation was continued for about 11 s, then suddenly stopped to record the free vibration of the building, as shown in Fig. 4.

**Table 2:** Location of sensors, measurement direction, and testing method of each measurement.

Day	Location of sensors	Measurement direction	Testing method
March 27^th^	Case 1	X	Microtremor measurement
		Y	
March 28^th^	Case 2	X	Microtremor measurement
		Y	
March 29^th^	Case 3	Y	Microtremor measurement
		Y	Human-powered excitation test

Note that the dataset of microtremor measurements, Case 1_X.xlsx, Case 1_Y.xlsx, Case 2_X.xlsx, Case 2_Y.xlsx and Case 3_Y.xlsx, contains the velocity data for five minutes, which were extracted from the 10- min data recorded while the elevator was off. Regarding to the dataset of the human-powered excitation test, Case 3_Y_free.xlsx, it contains the data recorded for one minute from before starting pushing walls to after decaying free vibration. As mentioned in Section 3, the data recorded during the human-powered excitation tests by sensor No. 3 exceeded the measurable amplitude range probably due to the initial inclination of the sensor; therefore, the data of sensor No. 3 is not contained in Case 3_Y_free.xlsx file.

Table 3 provides the temperature and wind speed recorded during the testing period at Vancouver International Airport (YVR) [6], which is about 10 km from the tested building and has similar weather conditions. The weather at the building site was partly cloudy and had no rain throughout the three days of testing.

**Table 3:** Temperature and wind speed recorded at Vancouver International Airport (YVR) during the testing period [6].

	March 27^th^		March 28^th^		March 29^th^	
Time	Temperature ( °C)	Wind speed ( km/h)	Temperature ( °C)	Wind speed ( km/h)	Temperature ( °C)	Wind speed ( km/h)
1:00 P.M	8.9	17	16.3	35	11.8	21
2:00 P.M	8.9	23	13.0	18	10.8	21
3:00 P.M	9.9	20	13.2	14	11.7	16
4:00 P.M	10.2	21	12.7	28	11.5	19
Average	9.5	20	13.8	24	11.5	19

## Ethics Statements

This work does not involve human subjects, animal experiments, or any data collected from social media platforms.

## CRediT authorship contribution statement

**Yuji Miyazu:** Methodology, Data curation, Visualization, Writing – review & editing. **Cristiano Loss:** Project administration, Conceptualization, Methodology, Writing – review & editing.

## Data Availability

Lateral vibration data of an actual 18-story timber-concrete hybrid building (Original data) (Mendeley Data).

## References

[1] Tokyo Sokushin Co., Ltd., Servo velocity-meter VSE-15. https://www.to-soku.co.jp/en/products/servo/pdf/vse15d_e.pdf, 2023 (accessed 16 July 2023).

[2] Tokyo Sokushin Co., Ltd., Portable vibration monitoring system SPC-52. https://www.to-soku.co.jp/en/products/portable/pdf/spc_52_e.pdf, 2023 (accessed 16 July 2023).

[3] D. Safarik, J. Elbrecht, W. Miranda, State of tall timber 2022, https://www.ctbuh.org/resources/papers/4530-Journal2022_IssueI_StateofTallTimber/TBIN.pdf, 2022 (accessed 16 July 2023)

[4] Miyazu Y., Loss C. (2023). Lateral vibration data of an actual 18-story timber-concrete hybrid building. Mendeley Data.

[5] Naturally:wood, Tallwood house storyboards. https://www.naturallywood.com/wp-content/uploads/brock-commons-storyboards_factsheet_naturallywood.pdf, 2016 (accessed 16 July 2023).

[6] Government of Canada, Historical climate data. https://climate.weather.gc.ca/historical_data/search_historic_data_e.html, 2023 (accessed 16 July 2023).

